# Prevalence and Risk Factors of Comorbid Type 2 Diabetes Mellitus in Adults With Severe Mental Disorders: A Retrospective Study

**DOI:** 10.31083/AP47535

**Published:** 2025-10-21

**Authors:** Jiao Hu, Xida Wang, Xuanwu Huang, Zhuozhuo Cheng, Huiling Ye, Haofeng Xu, Youping Wang

**Affiliations:** ^1^Department of General Practice, The First Affiliated Hospital, Guangzhou Medical University, 510120 Guangzhou, Guangdong, China; ^2^Department of Emergency Medicine, The Affiliated Brain Hospital, Guangzhou Medical University, 510370 Guangzhou, Guangdong, China; ^3^Guangdong Engineering Technology Research Center for Translational Medicine of Mental Disorders, 510370 Guangzhou, Guangdong, China

**Keywords:** severe mental disorders, type 2 diabetes mellitus, prevalence, risk factor

## Abstract

**Background:**

The purpose of this study was to investigate the incidence of comorbid type 2 diabetes mellitus (T2DM) and its associated risk factors in adult patients with severe mental disorders (SMD) who were admitted to the Affiliated Brain Hospital of Guangzhou Medical University.

**Methods:**

We conducted a retrospective analysis of the clinical data of adult patients with SMD admitted to our hospital. The research comprised 5964 adult inpatients with SMD. Data were collected from 1 January 2023, to 31 December 2023. The collected data encompassed demographic details, classifications of mental disorders, hospitalization records, concomitant conditions, and pertinent laboratory findings. We performed descriptive and inferential statistical analyses to assess the prevalence of T2DM and identify associated risk factors.

**Results:**

Patients with SMD had a 10.14% frequency of concurrent T2DM. In this patient cohort, our study found that age, body mass index (BMI), hypertension, triglyceride levels and apolipoprotein B levels were important risk factors for T2DM.

**Conclusion:**

The results show that T2DM is much more common in people with SMD and suggest that several clinical and demographic traits may increase the chance of developing this condition. Extensive screening and targeted treatments are necessary for this vulnerable group.

## Main Points

(1) The prevalence of type 2 diabetes mellitus (T2DM) among severe mental disorders (SMD) patients is 10.14%.

(2) The prevalence of T2DM among patients with schizophrenia is 15.19%, 8.39% among those with bipolar disorder, and 5.36% among those with major depressive disorder.

(3) Elevated body mass index (BMI), hypertension, increased triglycerides (TG), and high ApoB levels are identified as influencing factors for comorbid T2DM in SMD patients.

## 1. Introduction

Mental illness is classified into two main categories based on severity: mental illness and severe mental disorders (SMD). Mental illness includes all psychiatric diseases, whereas SMD refers to more severe forms of mental illness. SMD, such as schizophrenia, bipolar disorder, and major depressive disorder, are defined as mental, behavioral, or emotional disorders that result in considerable dysfunction and significantly hinder one or more aspects of daily life [1]. A research study indicates that mental illnesses are a major global health issue [2]. In China, over 240 million individuals are affected by mental illnesses, with more than 16 million experiencing SMD. Moreover, this figure continues to rise annually. SMD has consequently emerged as a substantial social and public health issue [3].

The escalating burden of SMD coincides with the rising incidence of chronic diseases, including diabetes mellitus (DM), which represents another significant global health concern. DM is a group of metabolic disorders characterized by chronic hyperglycemia, resulting from various etiological factors, including insulin deficiency and insulin resistance. Type 2 diabetes (T2DM), previously referred to as “noninsulin-dependent diabetes” or “adult-onset diabetes”, accounts for 90–95% of all DM [4]. According to the International Diabetes Federation (IDF), the prevalence and incidence of DM are increasing rapidly, with projections indicating that by 2045, the global number of individuals with DM will reach 783.2 million. The overwhelming majority of people with DM in 2021 have T2DM. Thus, the prevalence trends mainly reflect T2DM [5].

Individuals with mental illness have a markedly elevated prevalence of T2DM compared with the general population [6]. The prevalence of DM among individuals with mental illness varies from 1.26% to 50%, with a median of 13% [7]. Numerous meta-analytics indicate that around 10% of people with SMD have T2DM [8, 9, 10, 11]. The relationship between T2DM and SMD is complex, involving several contributing factors [12, 13]. Some behavioral and biological mechanisms link mental disorders with T2DM. Existing studies generally categorize these mechanisms into three types: behavioral, biological, and cognitive. (1) Behavioral factors: individuals with SMD are predisposed to unhealthy habits, including physical inactivity, smoking, and alcohol drinking, which markedly elevate the risk of acquiring T2DM. Moreover, pharmacological treatments for SMD may induce endocrine and metabolic adverse consequences, including weight gain and insulin resistance. (2) Biological factors: changes in biology, such as problems with the hypothalamic-pituitary-adrenal (HPA) axis and the sympathetic nervous system, may make it more likely that someone will get T2DM. (3) Cognitive variables: cognitive variables serve as possible mediators and encompass a reduction in cognitive capacity and focus, less responsiveness to enjoyable activities (anhedonia), chronic weariness, and a lack of drive. Furthermore, there is an interaction between cognitive and behavioral patterns. For example, an unhealthy lifestyle may lead to decreased attention or lack of motivation, which in turn may reduce the individual’s willingness to participate in health screenings and maintain an active lifestyle, ultimately having a negative impact on overall health [6].

Consequently, a principal objective of primary prevention should be to mitigate risk factors associated with T2DM in the prevention and management of these illnesses. Lifestyle modifications in patients with impaired glucose tolerance (IGT) have demonstrated efficacy in postponing the onset of T2DM [14]. Consequently, comprehending and recognizing the risk factors for T2DM coexisting with SMD is crucial.

Multiple studies have documented the prevalence and risk factors of T2DM among individuals with SMD. Yang *et al*. [12] found that the prevalence of T2DM among adult inpatients with mental illnesses in Beijing was 10.75%. A study conducted in Shanghai on persons with SMD revealed that 65.55% (413 out of 630) demonstrated decreased glucose metabolism [15]. Nevertheless, a lack of relevant research persists in southern China. The present study analyzed data from 5964 adult inpatients with severe mental disorders at the Affiliated Brain Hospital of Guangzhou Medical University in 2023 to evaluate the prevalence and identify risk factors for T2DM in this population. The aim was to identify high-risk traits associated with T2DM in hospitalized patients with SMD, enabling early diagnosis, intervention, and management to improve their quality of life.

## 2. Methods

### 2.1 Research Participants and Data Collection

This study is a retrospective analysis designed to evaluate the prevalence and risk factors of concomitant T2DM in inpatients with SMD at the Affiliated Brain Hospital of Guangzhou Medical University. Data were gathered from 1 January 2023, to 31 December 2023.

The inclusion criteria were inpatients diagnosed with SMD, namely schizophrenia, bipolar disorder, and major depressive disorder, who were discharged within the research period. The exclusion criteria encompassed patients with duplicate data resulting from readmission within 1 year, as only data from their original hospitalization were preserved to prevent duplication. Furthermore, those under 18 years of age and those with ambiguous diagnoses or not categorized according to the 10th edition of the International Classification of Diseases (ICD-10) were omitted.

The data-collecting process entailed retrieving pertinent information from the hospital’s electronic medical record system. This approach ensured the meticulousness and accuracy of the records. We anonymized all data to safeguard patient privacy and guarantee data security. We identified essential variables, encompassing demographic information such as age, gender, marital status, educational level, and body mass index (BMI); comorbidities including T2DM, hypertension, and fatty liver disease; as well as laboratory test results such as uric acid (UA), total cholesterol (TC), triglycerides (TG), low-density lipoprotein cholesterol (LDL-C), high-density lipoprotein cholesterol (HDL-C), and apolipoprotein B (ApoB).

### 2.2 Statistical Analysis

The socio-demographic data and clinical characteristics of patients with and without T2DM were described. Categorical variables were expressed as frequencies and percentages, and the chi-squared (χ^2^) test was used to compare rates between groups. The Kolmogorov–Smirnov one-sample test was used to examine whether each parameter obeyed normal distribution. Data that did not conform to normal distribution were shown as median (M) (Q1, Q3) and tested by the Mann–Whitney U-test. Multiple regression analysis was used to identify the significant factor related to T2DM in patients with SMD by calculating an odds ratio (OR) and 95% confidence interval (CI). The socio-demographic, clinical characteristics, and laboratory indicators were included in the multiple regression analysis according to the results of the univariate analysis.

All analyses were performed using SPSS version 25.0 software (IBM Corp., Chicago, IL, USA). Mapping was performed using R version 4.4.2 software (R Foundation for Statistical Computing, Vienna, Austria). All tests were two-tailed and statistical significance was defined at *p* < 0.05.

## 3. Results

### 3.1 General Characteristics of Study Participants

A total of 5964 adult inpatients with SMD were included in the study, with a median age of 37.00 (26.00, 58.00) years; among these, 2833 (47.50%) were males and 3131 (52.50%) were females. Of the total inpatients, 2182 (36.59%) were married and 3782 (63.41%) were not currently married, including those that were unmarried, divorced, widowed, or in other situations, and 3956 (66.33%) were educated to below bachelor’s degree level and 2008 (33.67%) had a bachelor’s degree or above.

### 3.2 Prevalence of T2DM in Adults With SMD

Of the 5964 inpatients with SMD, 605 had a T2DM diagnosis, resulting in an overall prevalence of 10.14% (605/5964). The prevalence of T2DM among the 2429 individuals with schizophrenia was 15.19% (369/2429). Out of the 1538 individuals diagnosed with bipolar disorder, 129 also received a T2DM diagnosis, resulting in an 8.39% prevalence rate (129/1538). Ultimately, 107 of the 1997 individuals diagnosed with severe depression also had T2DM, yielding a prevalence rate of 5.36% (107/1997).

### 3.3 Comparison Characteristics Between non-T2DM and T2DM Groups in Individuals With SMD

As shown in Table 1, there were statistically significant differences between the T2DM and non-T2DM groups in terms of age, non-marital status, educational attainment, BMI, types of SMD, hypertension, fatty liver, TG, HDL-C, ApoB, and TC (*p* < 0.001).

**Table 1.** **Comparison of Clinical Characteristics Between Non-T2DM and T2DM Groups in Individuals with Severe Mental Disorders**.

Variable			Total (n = 5964)	Non-T2DM (n = 5359)	T2DM (n = 605)	*p*
Sociological information						
	Age (year), M (Q_1_, Q_3_)		37.00 (26.00, 58.00)	35.00 (25.00, 54.00)	62.00 (54.00, 70.00)	<0.001^a^
	Gender, n (%)					0.905^b^
		Female	3131 (52.50)	2812 (52.47)	319 (52.73)	
		Male	2833 (47.50)	2547 (47.53)	286 (47.27)	
	Non-marital status, n (%)					<0.001^b^
		No	2182 (36.59)	1898 (35.42)	284 (46.94)	
		Yes	3782 (63.41)	3461 (64.58)	321 (53.06)	
	Educational attainment, n (%)					<0.001^b^
		<Bachelor’s degree	3956 (66.33)	3440 (64.19)	516 (85.29)	
		≥Bachelor’s degree	2008 (33.67)	1919 (35.81)	89 (14.71)	
Clinical characteristics						
	BMI (kg/m^2^), M (Q_1_, Q_3_)		22.49 (19.85, 25.54)	22.38 (19.69, 25.39)	23.61 (21.23, 26.56)	<0.001^a^
	Type of SMD, n (%)					<0.001^b^
		Schizophrenia	2429 (40.73)	2060 (38.44)	369 (60.99)	
		Bipolar Disorder	1538 (25.79)	1409 (26.29)	129 (21.32)	
		Depressive Disorder	1997 (33.48)	1890 (35.27)	107 (17.69)	
	Hypertension, n (%)					<0.001^b^
		No	5182 (86.89)	4861 (90.71)	321 (53.06)	
		Yes	782 (13.11)	498 (9.29)	284 (46.94)	
	Fatty Liver, n (%)					<0.001^b^
		No	5128 (85.98)	4646 (86.70)	482 (79.67)	
		Yes	836 (14.02)	713 (13.30)	123 (20.33)	
Laboratory indicators						
	UA (µmol/L), M (Q_1_, Q_3_)		378.00 (306.00, 459.00)	378.00 (306.00, 458.00)	383.00 (307.00, 462.00)	0.518^a^
	LDL-C (mmol/L), M (Q_1_, Q_3_)		2.59 (2.10, 3.17)	2.60 (2.11, 3.18)	2.54 (2.05, 3.14)	0.119^a^
	TG (mmol/L), M (Q_1_, Q_3_)		1.07 (0.78, 1.53)	1.04 (0.76, 1.48)	1.34 (0.99, 1.92)	<0.001^a^
	HDL-C (mmol/L), M (Q_1_, Q_3_)		1.27 (1.09, 1.49)	1.28 (1.10, 1.50)	1.18 (1.00, 1.37)	<0.001^a^
	ApoB (g/L), M (Q_1_, Q_3_)		0.88 (0.72, 1.07)	0.88 (0.71, 1.06)	0.98 (0.80, 1.20)	<0.001^a^
	TC (mmol/L), M (Q_1_, Q_3_)		4.40 (3.80, 5.20)	4.50 (3.90, 5.20)	4.30 (3.60, 5.00)	<0.001^a^

M, Median; Q_1_, 1st quartile; Q_3_, 3rd quartile; T2DM, Type 2 Diabetes Mellitus; SMD, severe mental disorders; BMI, body mass index; UA, uric acid; LDL-C, low-density lipoprotein cholesterol; TG, triglycerides; HDL-C, high-density lipoprotein cholesterol; ApoB, apolipoprotein B; TC, total cholesterol. ^a^Mann-Whitney test, ^b^chi-squared test.

### 3.4 Univariate and Multivariate Logistic Regression Analysis of Comorbid T2DM in Adults 

**Fig. 1.**
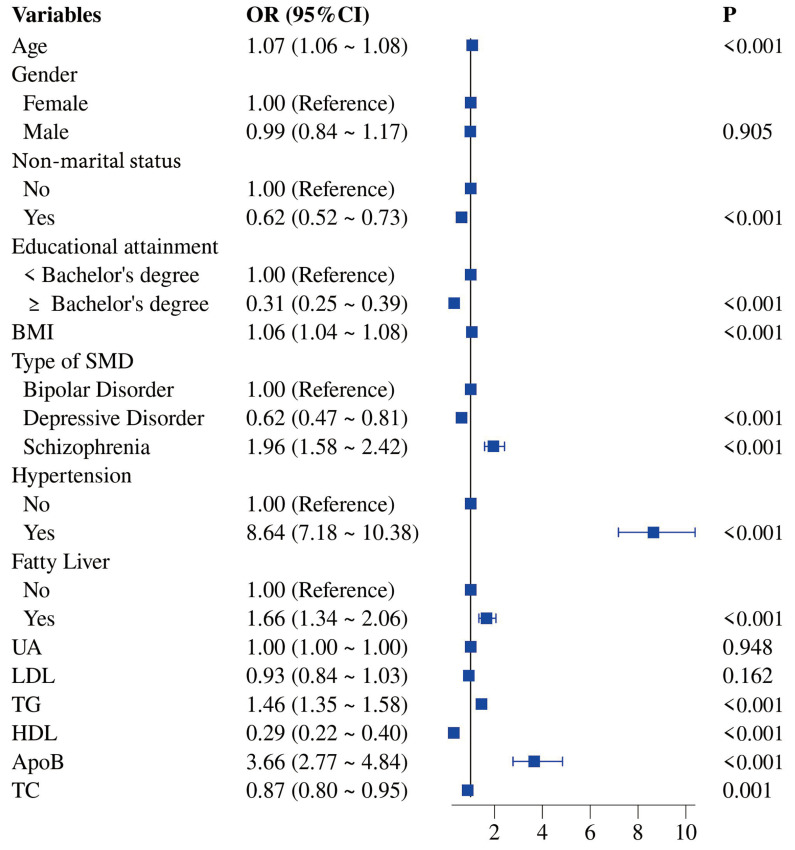
**Forest Plot of Univariate Logistic Regression Analysis for Risk Factors of T2DM in Adults with SMD**.

**Fig. 2.**
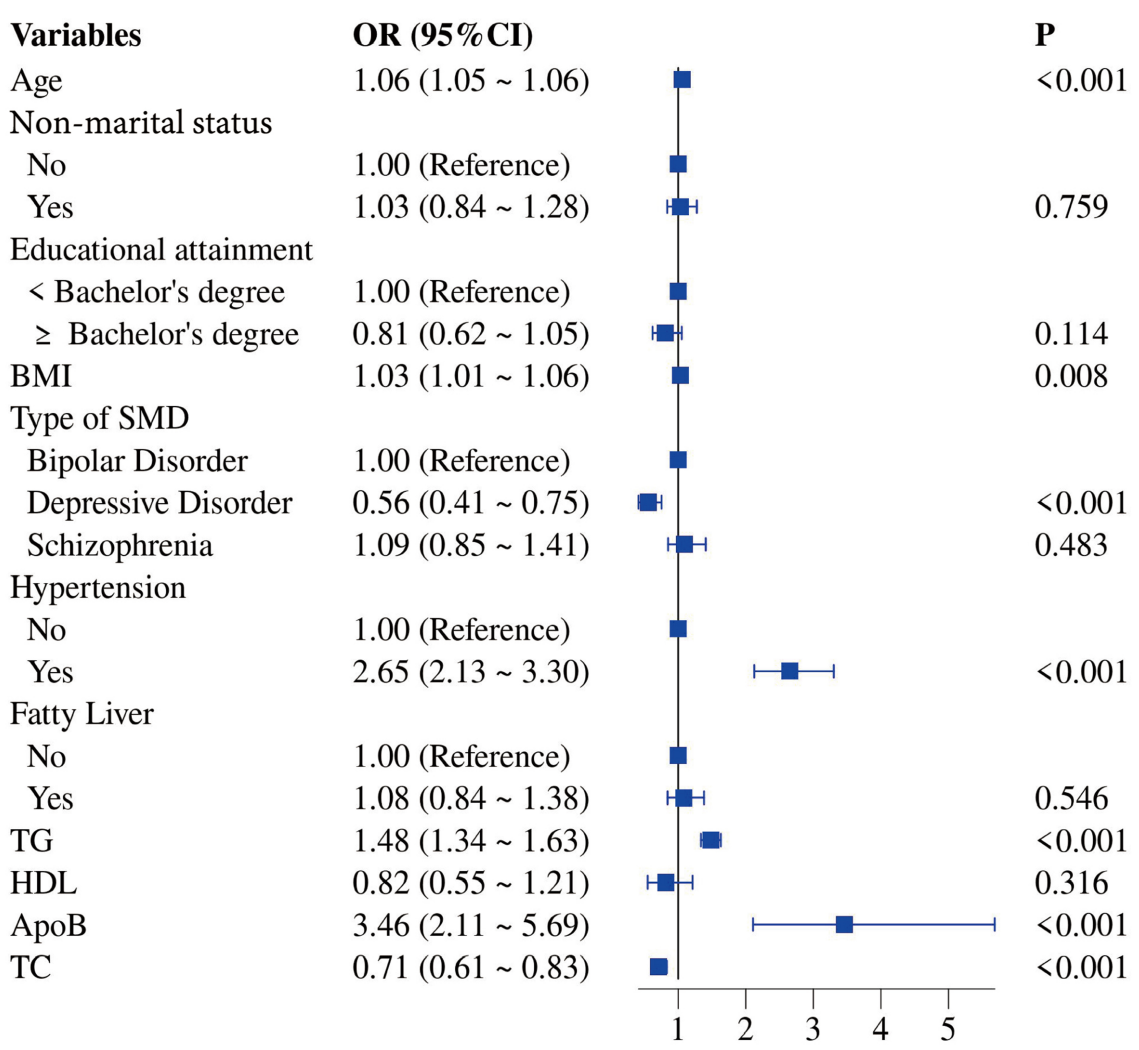
**Forest Plot of Multivariate Logistic Regression Analysis for Risk Factors of T2DM in Adults with SMD**.

Initially, we performed univariate logistic regression analysis to first evaluate the association of T2DM and 14 variables (age, gender, non-marital status, educational attainment, BMI, type of SMD, hypertension, fatty liver, UA, LDL-C, TG, HDL-C, ApoB, TC) in SMD. Eleven significant variables (*p* < 0.001; Table 2) were included in the multivariate logistic regression analysis for further screening. We identified seven variables as predictors of the development of T2DM in adults with SMD. Advanced age, increased BMI, hypertension, raised TG, and high ApoB were associated with an increased risk of T2DM in adult SMD patients. Major depressive disorder and increased TC were associated with a reduced risk of T2DM in adults with SMD (Figs. 1,2).

**Table 2.** **Results of univariate and multivariate logistic regression analyses**.

Variable		Univariate logistic regression		Multivariate logistic regression	
		*p*	OR (95% CI)	*p*	OR (95% CI)
Age		<0.001	1.07 (1.06~1.08)	<0.001	1.06 (1.05~1.06)
Gender					
	Female		1.00 (Reference)		
	Male	0.905	0.99 (0.84~1.17)		
Non-marital status					
	No		1.00 (Reference)		1.00 (Reference)
	Yes	<0.001	0.62 (0.52~0.73)	0.759	1.03 (0.84~1.28)
Educational attainment					
	<Bachelor’s degree		1.00 (Reference)		1.00 (Reference)
	≥Bachelor’s degree	<0.001	0.31 (0.25~0.39)	0.114	0.81 (0.62~1.05)
BMI		<0.001	1.06 (1.04~1.08)	0.008	1.03 (1.01~1.06)
Type of SMD					
	Bipolar Disorder		1.00 (Reference)		1.00 (Reference)
	Depressive Disorder	<0.001	0.62 (0.47~0.81)	<0.001	0.56 (0.41~0.75)
	Schizophrenia	<0.001	1.96 (1.58~2.42)	0.483	1.09 (0.85~1.41)
Hypertension					
	No		1.00 (Reference)		1.00 (Reference)
	Yes	<0.001	8.64 (7.18~10.38)	<0.001	2.65 (2.13~3.30)
Fatty liver					
	No		1.00 (Reference)		1.00 (Reference)
	Yes	<0.001	1.66 (1.34~2.06)	0.546	1.08 (0.84~1.38)
UA		0.948	1.00 (1.00~1.00)		
LDL-C		0.162	0.93 (0.84~1.03)		
TG		<0.001	1.46 (1.35~1.58)	<0.001	1.48 (1.34~1.63)
HDL-C		<0.001	0.29 (0.22~0.40)	0.316	0.82 (0.55~1.21)
ApoB		<0.001	3.66 (2.77~4.84)	<0.001	3.46 (2.11~5.69)
TC		0.001	0.87 (0.80~0.95)	<0.001	0.71 (0.61~0.83)

OR, odds ratio; CI, confidence interval.

The principal findings of the univariate logistic regression analysis indicate that age, non-marital status, educational attainment, BMI, type of SMD, hypertension, fatty liver, TG, HDL-C, ApoB, and TC are associated with the occurrence of T2DM.

Risk factors identified include advanced age, increased BMI, hypertension, raised TC and high ApoB. Protective factors include major depressive disorder and increased TC.

## 4. Discussion

The prevalence of comorbid T2DM is higher in patients with severe mental illness compared with the general population. In the present study, the prevalence of T2DM individuals with SMD was 10.14%, with a prevalence rate of 15.19% among schizophrenic patients, 8.39% among bipolar disorder patients, and 5.36% among patients with major depressive disorder. These results are consistent with previous investigations. The repercussions of T2DM in persons with SMD are more pronounced [16, 17]. The prevalence of T2DM in individuals with mental illness varies according to the specific mental disorder, ranging from around 5% to 22%, according to an international comprehensive meta-analysis [7]. Das-Munshi *et al*. [18] discovered that the prevalence of T2DM comorbid with SMD was 16.00%. Liu and Miu [19] identified a T2DM prevalence rate of 18.29% among community patients with SMD. Yang *et al*. [12] conducted research on adult mental inpatients in Beijing, revealing a T2DM prevalence rate of 10.75% and an 11.63% prevalence of patients with schizophrenia. Numerous studies conducted in China on schizophrenic patients have revealed a prevalence rate of approximately 13% for T2DM [20, 21]. Furthermore, one study indicates that female schizophrenic patients have a greater prevalence of T2DM [22]. However, Li *et al*. [15] found that among 630 patients with SMD, the prevalence of abnormal glucose metabolism was as high as 65.55% (413 cases).

Numerous risk factors for T2DM are present in persons with SMD. This investigation found advanced age, increased BMI, hypertension, raised TG, and high ApoB to be risk factors for comorbid T2DM in individuals with SMD. In contrast, major depressive disorder and increased TC may act as protective factors. Notably, gender did not show a significant difference in the prevalence of T2DM among individuals with SMD, which is consistent with some previous research findings [13, 22, 23]. Research suggests that gender may not significantly influence the increased frequency of T2DM, implying the presence of clinical confounding variables [8].

Advanced age is a recognized risk factor for T2DM, especially in those with SMD [14]. The combined effects of physiological aging and chronic mental diseases may increase the risk of T2DM due to the longer duration of SMD, as evidenced by previous studies [9, 11, 24].

Individuals with schizophrenia exhibit a greater incidence of T2DM, and both genetic and epidemiological research have shown a correlation between schizophrenia and T2DM. Shared pathophysiological mechanisms and genetic factors may contribute to the co-occurrence of schizophrenia and T2DM, while antipsychotic medications are known to increase the risk of developing T2DM [25, 26, 27]. The present study found no significant association between schizophrenia and T2DM. This may be attributable to the use of bipolar affective disorder as a reference group in the statistical analysis. Bipolar disorder is known to be associated with an increased risk of insulin resistance or even a bidirectional relationship with T2DM [28, 29]. Antidepressant medications may have influenced the lower incidence of T2DM among patients with severe depression in this study. T2DM may be partly influenced by the effects of antidepressant medications [30, 31]; other contributing factors include unhealthy diet, sedentary lifestyle, smoking, and reduced physical activity [32]. Additionally, autonomic dysfunction, systemic inflammation, and dysregulation of the HPA axis in depression contribute to the development of T2DM [33]. Nonetheless, this represents a nuanced and relatively obscure facet of the association between depression and T2DM, and the evidence remains inconclusive.

Hypertension is an additional recognized risk factor for comorbid T2DM in patients with SMD. A significant number of these patients maintain sedentary lifestyles, adversely affecting their blood pressure and thereby increasing the risk of T2DM. Suboptimal eating practices and diminished physical activity frequently result in weight gain, thereby elevating the incidence of T2DM among individuals with mental problems [21, 22, 24].

High TG and elevated ApoB are major lipid markers associated with an increased risk of T2DM in patients with SMD [34]. ApoB, predominantly located in LDL-C, acts as a biomarker indicative of lipid concentrations. Elevated levels of TG and ApoB are indicative of dyslipidemia, which is a significant contributor to the development of T2DM [35]. Despite the identification of associations between lipid-related genetic polymorphisms and T2DM, the precise genetic processes remain unclear [36]. Our findings indicate that hypercholesterolemia may have a protective benefit in SMD patients, contrasting with prior research that generally identified elevated cholesterol as a risk factor for T2DM [37]. Research has shown that total cholesterol levels are not elevated in individuals with SMD [38]. The intricate link between biomarkers and illness risk in various patient populations highlights the necessity of personalized therapy options. Obesity is a significant risk factor, particularly for individuals with SMD, as it increases the likelihood of developing T2DM [39, 40]. Unhealthy behaviors, including sedentary lives and inadequate eating practices, frequently lead to obesity [6]. Additional risk factors influencing this group may encompass ethnicity, employment, smoking history, utilization of psychiatric drugs, length of SMD, familial history of T2DM, concomitant cardiovascular disease, and socioeconomic status. These data demonstrate diversity in recognized risk variables across both local and foreign investigations.

A main constraint of our analysis is the reliance on a single-center data source. Findings from one institution may not adequately reflect the broader population of SMD patients in China, given the significant variability in regional healthcare practices, demographic factors, and social determinants of health. Therefore, we must carefully interpret our findings and limit their applicability to the wider Chinese population. Further multicenter studies involving larger and more diverse populations are necessary to validate and expand upon our findings. The retrospective nature of the data and the available information constrained the selection of variables in this study. We incorporated pertinent variables from existing literature but selected some based on availability rather than robust evidence, thereby constraining the investigation of potential contributors. Prospective studies with expanded data collection methodologies could effectively address this issue. The complexity of the diagnostic criteria for mental disorders and T2DM may have affected the accuracy of the results. The trial lacks sufficient long-term follow-up data, limiting the ability to evaluate disease progression and patient outcomes.

## 5. Conclusion

Individuals with SMD demonstrate a significant prevalence of comorbid T2DM, especially in older adults with elevated BMI, hypertension, increased TG, and high ApoB levels. Consequently, in the management of patients with SMD, it is imperative to concentrate on these high-risk populations, perform early screenings, and apply preventive strategies to mitigate the occurrence of T2DM and its related health hazards.

## Availability of Data and Materials

The data sets produced and examined during this study can be obtained from the corresponding author upon reasonable request.
